# Head and Neck Dermatitis in Atopic Dermatitis: A Narrative Review of Pathogenesis, Clinical Challenges, and Therapeutic Strategies

**DOI:** 10.3390/antib14040104

**Published:** 2025-12-05

**Authors:** Giuseppe Lauletta, Cataldo Patruno, Claudio Brescia, Andrea Cosenza, Carolina D’Elia, Valentina Ventura, Emanuela Martina, Maddalena Napolitano

**Affiliations:** 1 Section of Dermatology, Department of Clinical Medicine and Surgery, University of Naples Federico II, 80128 Naples, Italy; claudio.brescia.1997@gmail.com (C.B.); andreacosenza.med@gmail.com (A.C.); carolinadelia26@gmail.com (C.D.); valentinaventura.salerno@gmail.com (V.V.); maddalena.napolitano@unina.it (M.N.); 2 Department of Medicine and Health Sciences “Vincenzo Tiberio”, University of Molise, 86100 Campobasso, Italy; cataldopatruno@libero.it; 3 Dermatology Clinic, Department of Clinical and Molecular Sciences, Polytechnic Marche University, 60121 Ancona, Italy; emamartina@gmail.com

**Keywords:** atopic dermatitis, head and neck dermatitis, dupilumab, tralokinumab, lebrikizumab, JAK inhibitors

## Abstract

**Background**: Head and neck dermatitis (HND) represents a challenging phenotype of atopic dermatitis (AD), often showing suboptimal response or paradoxical worsening during biologic therapy. **Objective**: To review the efficacy and safety of current systemic treatments for HND, with a focus on dupilumab, tralokinumab, lebrikizumab, and janus kinase (JAK) inhibitors. **Methods**: We conducted a narrative review of randomized controlled trials, post hoc analyses, and real-world studies assessing clinical outcomes in patients with moderate-to-severe AD involving the head and neck. Outcomes included Eczema Area and Severity Index (EASI) H&N subscore, erythema grade, patient-reported measures, and adverse events. **Results**: Dupilumab shows substantial efficacy for HND in both clinical trials and real-life studies; however, responses are often less pronounced than in other anatomical regions, and facial redness (FR) has emerged as a notable adverse event in up to 9% of patients. Tralokinumab and lebrikizumab demonstrate significant improvements in HND involvement, with low incidence of paradoxical reactions. JAK inhibitors, particularly upadacitinib, provide rapid and marked improvement in refractory cases and in patients developing FR during biologic therapy. **Conclusions**: Systemic therapy for HND should be individualized, balancing efficacy and tolerability. JAK inhibitors represent a valuable alternative in biologic-refractory phenotypes or in patients experiencing dupilumab-associated FR.

## 1. Introduction

Atopic dermatitis (AD) is a chronic, relapsing inflammatory skin disease that affects up to 20% of children and 10% of adults worldwide [1,2]. Some children experience a resolution of symptoms over time and may no longer exhibit AD [3]. For others, AD can continue into adulthood and persist throughout their lives [3]. Adults may also develop AD later in life. The factors influencing the age of onset, progression, remission, and persistence of disease remain unclear and are the focus of ongoing research [3].

AD is a heterogeneous condition, presenting a broad clinical spectrum that may evolve over time [4]. The clinical features of AD include eczematous and itchy lesions, with a distribution pattern that varies depending on the patient’s age [1,4]. Infants typically present with acute, exudative lesions around the mouth, which can spread to the rest of the face, the extensor surfaces, and the trunk. In children over 2 years, dry skin, lighter-coloured erythema, and lichenified papules and plaques typically develop in flexural areas, hands, and feet [5,6]. In adolescents and adults, eczema often becomes more lichenified and typically involves the hands, neck, eye area, and flexures [7].

The head and neck region may be affected across all age groups, often presenting significant therapeutic challenges. Head and neck dermatitis (HND), recognized as a subtype of AD, predominantly involves the seborrheic areas—namely the head, face, neck, and upper trunk [8,9]. A clear distinction between HND and seborrheic dermatitis is clinically relevant, as these two conditions may overlap in distribution but differ in etiology and cutaneous features. HND represents an atopic dermatitis phenotype characterized by impaired barrier function, type 2-skewed inflammation, and frequent Malassezia-associated immune responses [8,9]. In contrast, seborrheic dermatitis is primarily a chronic papulosquamous disorder linked to sebaceous activity, Malassezia proliferation, and innate immune dysregulation. Clinically, seborrheic dermatitis presents with greasy scales and erythema confined to seborrheic areas (scalp, nasolabial folds, eyebrows), while HND typically shows eczematous lesions, lichenification, xerosis, and a personal or familial atopic background [8,9]. Awareness of these distinctions is crucial, as management strategies differ and misclassification may contribute to apparent therapeutic refractoriness in the head and neck region. Prevalence rates indicate that HND affects up to 36% of adult AD patients and 79% of pediatric cases [8,10,11]. In infants, lesions commonly occur on the cheeks and anterior neck [12]. Among children, the flexural surface of the neck is typically involved, with facial involvement observed less frequently [12]. Adolescents and adults most often exhibit orbital eczema [12]. Notably, while anterior neck involvement is frequent, cutaneous manifestations in this area are generally more severe compared to those observed on the posterior neck [12].

HND is linked to a higher health-related quality of life burden in both adults and children when compared with AD affecting other body areas [13]. Evidence indicates that involvement of the head, neck, and facial regions has a larger effect on quality of life than either objective severity scores or the total body surface area affected [13].

This narrative review seeks to provide a comprehensive examination of HND within the broader framework of pathogenesis, with particular attention to the varying impacts of biological therapies and small-molecule agents on this specific AD subtype.

## 2. Materials and Methods

A literature search was performed across various databases including PubMed, Embase, Medline, and Web of Science, Scopus covering articles published from January 2018 to June 2025 using keywords such as “HND,” “facial AD,” “facial eczema,” “head and neck eczema,” “regional AD,” “HND treatments,” “face and neck AD,” and “face eczema therapies.” All identified articles were evaluated for relevance, and their references were reviewed to locate additional sources. Data from the selected articles were compiled and analyzed for this narrative review. Inclusion criteria were original research articles, clinical trials, systematic reviews, or meta-analyses; studies focused on systemic treatments for moderate to severe AD. Topical therapies were not included in this review because HND requiring medical attention is most frequently observed in patients with moderate-to-severe AD who have already failed or insufficiently responded to optimized topical regimens. For this reason, and in line with our predefined inclusion criteria, we focused exclusively on systemic treatments.

Only English-language research on HND was included; studies in other languages were excluded unless an English version was available. The reference lists of relevant articles were also screened to identify additional studies.

## 3. HND Pathogenesis

The AD pathogenesis involves both genetic predispositions and environmental influences, whose combined effects precipitate cutaneous inflammation [1]. Three principal mechanisms are implicated: (1) skin barrier disruption; (2) immune system dysregulation; and (3) skin microbiome alterations [1]. HND represents a clinical variant of AD that shares this underlying pathogenetic basis [12]. Nonetheless, the current literature indicates additional factors contributing to the prevalence of lesions in these regions, including heightened exposure to airborne and contact allergens as well as the modulation of inflammatory responses by Malassezia species [1,12].

### 3.1. Skin Barrier Disruption

Filaggrin (Filament Aggregating Protein—FLG) is a prominent epidermal protein that plays a pivotal role in the pathogenesis of AD and allergic diseases. Its precursor protein profilaggrin is a highly phosphorylated, functionally inactive polymer [14]. Profilaggrin is stored in keratohyalin granules in the stratum granulosum. Profilaggrin is dephosphorylated and cleaved by endo-proteases into 10- to 12-filaggrin monomers, which bind to keratin filaments [14,15]. FLG monomers are deamimated and degraded by proteases, releasing a pool of hygroscopic amino acids and derivatives of the natural moisturizing factors (NMFs) [15].

FLG plays an essential role in maintaining the structure and function of the stratum corneum (SC), conferring biochemical properties that enable resilience against environmental threats and maintain homeostasis [16]. As mentioned, FLG is metabolized into NMFs, which can be measured in the SC. NMF levels are low at birth and increase with age [17]. Cheek SC, compared with elbow flexure and nasal tip, has the lowest NMF in the first year of life and is the slowest to reach stable levels, with immature corneocytes [17]. Regional differences in NMF levels, corneocyte envelope immaturity and protease activities may help explain why infantile AD most often initially affects the cheeks [17].

Furthermore, loss-of-function (LoF) mutations in FLG represent the key genetic susceptibility factor for AD development, with recent advances in genomics and genetics broadening our understanding of FLG mutations and identifying other FLG-related candidate genes implicated in disease development [16]. Meta-analyses have estimated the odds ratio for LoF mutations FLG gene and AD to be between 3.12 and 4.78 [16,18]. Additionally, maternal FLG mutations have been demonstrated to increase the risk of AD independently of whether the mutation is inherited [16,18].

AD associated with FLG mutations has a distinct phenotype (AD FLG) characterized by earlier onset and persistent disease, increased severity, predilection to the cheeks and hands, palmar hyperlinearity, increased risk of eczema herpeticum and staphylococcal infection, increased risk of asthma (including treatment-resistant asthma), and increased risk of food allergy as well as allergic sensitization [18]. Furthermore, subjects with AD FLG experience higher rates of hospitalization and incur greater long-term medication expenses [16].

### 3.2. Malassezia

The microbiome is increasingly recognized as a crucial factor in AD, with distinct microbiome profiles correlating with disease presence and severity [19,20]. To date, bacterial contributions—particularly from Staphylococcus aureus—have been the most thoroughly investigated and acknowledged [20,21]. Nevertheless, accumulating evidence also implicates fungi, especially *Malassezia* spp., in HND involving both T helper (Th)2 and Th17 immune pathways [22].

Malassezia represents the predominant genus among cutaneous fungi, with sebaceous regions of the skin providing optimal conditions for its proliferation [23]. Colonization by Malassezia typically increases throughout the first year of life, stabilizes in childhood, and then escalates markedly at puberty, corresponding to rising androgen levels and associated increases in sebum production [24].

Representation of specific *Malassezia* spp. varies with demographic, micro-environmental (i.e., body location), and macro-environmental (i.e., geographic locations and ethnicities) factors [25]. Regarding age and body part, *M. globosa* is more frequently cultured from younger patients and along the scalp and forehead, while M. *sympodialis* is more commonly found in adult patients and along the skin of the back. This may be related to changes in skin lipid and pH profiles with age [25,26].

The diversity and relative abundance of Malassezia species may be related to AD [26]. Among AD patients, *M. globosa* and *M. restricta* appear to be more prevalent than M. *sympodialis* [27]. Furthermore, alterations in mycobiome composition may correlate with disease severity [27,28]. Patients with mild-to-moderate HND demonstrate a higher abundance of *M. restricta* compared to *M. globosa* within lesional skin, mirroring findings observed in between-flare AD skin [29]. Conversely, severe HND lesions display approximately equal proportions of these two species [29]. Therefore, an increased ratio of *M. globosa* to *M. restricta* may indicate progression towards greater disease severity in HND [29]. Notably, in AD patients, Malassezia cultures are less frequently positive and exhibit lower colony-forming unit counts in lesional skin compared to non-lesional areas [24,25]. This phenomenon may be attributed to the dysregulated yet inherently microbicidal immune environment associated with AD, which could actively inhibit Malassezia proliferation [24,25]. Episodes of severe HND may be precipitated by transient increases in Malassezia colonization that amplify the host immune response [24,25].

Furthermore, AD patients exhibit a skin surface pH that is up to 0.9 pH units higher than the mean healthy control skin pH of 5.24 [26]. Alkaline pH not only fosters Malassezia growth but also increases Malassezia antigen production [26]. These antigens can trigger skin inflammation through Th17 or Th2 pathways [30]. In the Th17 pathway, these antigens prompt neutrophils or monocytes to release IL-23, leading to Th17 or innate lymphoid cells secreting IL-17 and an overactive antifungal response; this is supported by increased Malassezia-specific Th17 memory T cells in AD patients [30,31]. In the Th2 pathway, dendritic cells present fungal antigens to Th2 cells, prompting IL-4 and IL-13 secretion, B-cell class switching, IgE production, and an allergic response. Higher Malassezia-specific IgE levels are linked to more severe HND [30,31]. It is important to clarify that current evidence does not support the concept of “Malassezia overgrowth” or active fungal proliferation as the direct cause of HND. As demonstrated by Chong et al. [25] and Glatz et al. [31], Malassezia detection rates may even be lower in AD lesions despite strong antigen-specific immune responses. The pathogenic contribution of Malassezia in HND therefore appears to be immunologic rather than microbiologic, which also explains why antifungal monotherapy often results in only partial improvement.

### 3.3. Cytokines Profile

The onset of acute AD lesions is typically characterized by significant upregulation of antimicrobial peptides along with increased expression of Th2 and Th22 cytokines [32]. As the disease progresses to a chronic stage, elevations in the Th2 and Th22 pathways persist, accompanied by notable increases in Th1 and Th17 markers among patients with chronic AD [32]. Notably, variations in cytokine profiles and clinical phenotypes have been documented among AD patients across different ethnicities and age groups, underscoring the complexity of AD pathogenesis [33]. Nevertheless, comprehensive analyses comparing cytokine and chemokine profiles at various anatomical sites—such as the face, extremities, and trunk—within individuals affected by AD remain limited.

Kido—Nakahara M et al. recently conducted a study comparing the expression levels of Th2, Th1, and Th17 cytokines and chemokines in the stratum corneum of skin lesions located on the forehead and abdomen in patients with AD, as well as in normal skin from control subjects, utilizing tape stripping methodology [34]. The study demonstrated that the expression profiles of these cytokines and chemokines varied not only between anatomical sites in AD patients but also among healthy controls [34]. The expression patterns of Th2 cytokines and chemokines vary by anatomical site within individuals affected by AD [34]. Specifically, IL-13 and IL-33 demonstrate higher expression levels in the trunk compared to the face, whereas IL-4 and IL-31 show elevated expression in the facial region relative to the trunk [34]. No significant differences were observed in TARC and TSLP expression in the stratum corneum between the forehead and abdomen. In contrast, Th1 and Th17 cytokine and chemokine levels are generally higher in the forehead than in the abdomen [34].

These regional disparities in stratum corneum cytokine and chemokine profiles may influence treatment responsiveness in AD.

## 4. HND Management

Systemic therapy plays a central role in the management of moderate-to-severe HND, particularly in patients who show insufficient response to topical agents or develop paradoxical reactions during biologic treatment. The following sections summarize current therapeutic options, including biologics and JAK inhibitors. A comparative overview of these treatments is provided in Table 1.

### 4.1. Dupilumab

Dupilumab, a fully human monoclonal antibody targeting the IL-4 receptor alpha subunit (IL-4Rα), inhibits the signalling of both IL-4 and IL-13 and represents the first biologic approved for the treatment of moderate-to-severe AD [44]. Clinical trials and real-world data have consistently shown that dupilumab leads to significant improvements in disease severity, pruritus, sleep quality, and overall quality of life [44,45,46,47]. Its safety profile, particularly in terms of long-term use, has further contributed to its widespread adoption in clinical practice [47]. While dupilumab’s efficacy has been extensively documented in generalized AD, increasing attention has been directed toward its role in treating site-specific phenotypes, particularly HND [48]. Although dupilumab is generally effective in this anatomical site, a subset of patients presents with either suboptimal response or paradoxical worsening, including new-onset facial erythema or dermatitis localized to the head and neck during treatment [49]. These clinical observations have sparked interest in understanding the underlying mechanisms, identifying predictive factors, and optimizing therapeutic strategies for this difficult-to-treat localization. The LIBERTY AD CHRONOS study, a phase 3, randomized, double-blind, placebo-controlled trial, originally demonstrated the long-term efficacy and safety of dupilumab in combination with topical corticosteroids (TCS) over 52 weeks in adults with moderate-to-severe AD [45]. In a post hoc analysis published by Blauvelt et al., the investigators specifically assessed the effectiveness of dupilumab on individual clinical signs of AD (erythema, infiltration/papulation, excoriation, and lichenification) across distinct anatomical regions, with a particular focus on the head and neck (H&N) area [35]. A total of 421 patients were included in this sub-analysis, of whom 106 received dupilumab 300 mg every two weeks plus TCS, and 315 received placebo plus TCS [35]. Disease severity was evaluated using the individual components of the Eczema Area and Severity Index (EASI), providing a region-specific quantification of the four core signs of AD. The results demonstrated that dupilumab significantly reduced all four clinical signs in the H&N area, although the magnitude of improvement was slightly attenuated compared to other body regions [35]. For erythema, the least squares (LS) mean percentage reduction from baseline at week 52 in the dupilumab group was –65.1%, corresponding to a placebo-corrected difference of –30.0%. By comparison, the same reduction in the lower extremities reached –34.8%, suggesting a modestly reduced response in the H&N region [35].

Similarly, for infiltration/papulation, excoriation, and lichenification, dupilumab led to statistically significant improvements, but the numerical response was lower in the H&N area compared to the trunk and limbs [35]. Clinical improvement was observed early during treatment. For erythema in the H&N, significant changes were already evident by week 2, and these effects were maintained through week 52, highlighting the durability of dupilumab’s response, even in this sensitive anatomical site [35]. Among the real-world studies investigating the use of dupilumab in patients with predominant H&N involvement, the retrospective cohort by Perego et al. represents one of the most comprehensive to date [36]. The study included 553 adult patients affected by moderate-to-severe AD with clinical involvement of the H&N region [36]. Patients were evaluated clinically at baseline and every four months during follow-up. Disease severity and control were assessed through multiple validated instruments, including the EASI score, the Pruritus Numerical Rating Scale (P-NRS), the Atopic Dermatitis Control Tool (ADCT), and the Dermatology Life Quality Index (DLQI) [36]. Importantly, patients were categorized based on their response in the H&N region into: (1) complete remission (CR): defined as EASI = 0 and P-NRS = 0, with no use of TCS or calcineurin inhibitors in the preceding 4 months; (2) intermittent AD: defined by alternating clinical activity and quiescence; (3) persistent AD: continuous involvement of the H&N over time [36]. At the 4-month follow-up, 30.7% of patients had achieved CR of the H&N lesions. This proportion increased to 45.1% at 12 months, and reached 55% after 36 months of treatment [36]. Among those who did not attain CR, the pattern of involvement shifted notably over time: at month 36, only 21.8% of patients displayed persistent disease, whereas 78.2% exhibited intermittent involvement, suggesting a dynamic and improving clinical trajectory in a substantial proportion of patients with continued treatment [36]. Quality of life (QoL) outcomes, as measured by DLQI and ADCT scores, were also analyzed and showed a statistically significant difference between responders and non-responders at all time points: at month 4, responders had a DLQI of 3.67 ± 4.07 versus 5.46 ± 5.15 in non-responders (*p* = 0.0001), and an ADCT of 4.94 ± 4.10 versus 6.45 ± 3.99 (*p* = 0.0001) [36]. At month 12, DLQI was 2.82 ± 3.63 vs. 4.11 ± 4.44 (*p* = 0.0005), and ADCT 3.43 ± 3.45 vs. 5.29 ± 4.32 (*p* = 0.0001). At month 24, DLQI was 2.10 ± 2.73 vs. 3.21 ± 3.22 (*p* = 0.0012), and ADCT 3.06 ± 3.59 vs. 4.64 ± 3.69 (*p* = 0.0002) [36]. Despite these encouraging results, real-world data also reveal a subset of patients in whom H&N lesions persist despite prolonged dupilumab therapy. In a nationwide prospective cohort study conducted in Denmark, Vittrup et al. followed 347 adult patients with moderate-to-severe AD treated with dupilumab for up to 104 weeks [50]. While significant improvements were observed in global disease severity, reflected by a reduction in median EASI score from 18.0 at baseline to 1.7 at week 104 (*p* < 0.0001), the proportion of patients reporting clinical involvement of the H&N region remained notably high throughout the treatment course. At baseline, 76% of patients had H&N involvement, which declined only modestly to 68% after two years of continuous therapy [50]. This finding contrasts with the more pronounced improvements observed in other anatomical sites, such as the hands (55% of patients reporting hand dermatitis at baseline and only 24% at 104 weeks follow-up), and highlights the relative therapeutic refractoriness of the H&N area [50]. Notably, while dupilumab demonstrated high drug survival (86% at 104 weeks) and durable improvements in EASI, POEM, DLQI, and P-NRS scores, the H&N region remained a site of incomplete resolution for a considerable subset of patients [50]. Moreover, facial redness (FR) or new-onset HDN during dupilumab treatment has emerged as a notable adverse effect [51]. In a large monocentric retrospective study, Napolitano et al. analyzed cutaneous adverse events (AEs) associated with dupilumab treatment in a real-life cohort of 916 adult patients with moderate-to-severe AD [52]. The study aimed to characterize the incidence, phenotype, timing, and management of dupilumab-associated cutaneous AEs, with a particular focus on the H&N region. Of the total cohort, 148 patients (16.2%) experienced at least one cutaneous AE [52]. Among these, FR emerged as one of the most frequent reactions, affecting 82 patients (8.95%) [52]. A more detailed diagnostic classification revealed that 73 patients (7.96%) had HND related to dupilumab, while 6 (0.65%) were diagnosed with allergic contact dermatitis (ACD) and 3 (0.32%) with alcohol-induced facial flushing (AIFF) [52].

Within the HND subgroup, 42 patients (4.58%) experienced a worsening of pre-existing facial dermatitis, whereas 31 (3.38%) developed new-onset HND under treatment. Most of these patients were male (52%) and had persistent AD (59.8%), with a mean time to onset of 4.14 months. Ocular involvement was present in 38.35% of HND cases, often as concurrent conjunctivitis [52].

Management of HND primarily involved topical calcineurin inhibitors (52%), TCS (37%), or both (6.8%). Systemic corticosteroids (16.4%) and systemic antifungals (6.8%) were used in selected cases [52]. Notably, 75.4% of patients responded to these interventions with substantial clinical improvement, while 24.7% (18/73) required dupilumab discontinuation due to persistent or severe HND [52]. Among the six patients with ACD, diagnoses were confirmed by patch testing, revealing sensitization to Kathon CG, sesquiterpene lactones (SLs), or Compositae allergens. All were male, and none discontinued dupilumab after allergen avoidance measures led to resolution [52]. Lastly, the three AIFF cases reported transient post-alcohol flushing, emerging on average 6.1 months after treatment initiation; no discontinuations were necessary [52].

These findings underscore the clinical heterogeneity of facial erythema during dupilumab therapy, ranging from exacerbated HND to distinct pathologies such as ACD or AIFF. While most cases are manageable with topical or supportive interventions, a subset of patients may require therapeutic adjustment or discontinuation, particularly when HND becomes persistent or recalcitrant [52].

Although not reported during clinical trials, dupilumab-associated FR has been described in real-life settings, with prevalence estimates ranging from 4% to 43.8%, affecting both adults and children [37]. The clinical onset is highly variable, ranging from a few weeks up to nine months after treatment initiation, and may present either as a worsening of preexisting facial dermatitis or as a new-onset eruption in patients without prior facial involvement [37]. Several hypotheses have been proposed to explain the underlying pathogenesis of DFR, pointing to a multifactorial etiology involving both immunologic shifts and local cutaneous microenvironmental factors. One of the most widely supported theories is Malassezia hypersensitivity, based on the predilection of the face and scalp for *Malassezia furfur* colonization, and the potential for Th17-skewed inflammation induced by IL-4/IL-13 blockade [37,53]. Another important mechanism is the unmasking of ACD [37,54]. Dupilumab’s suppression of Th2 pathways may allow Th1-mediated delayed hypersensitivity reactions to emerge more prominently. In this context, patch testing has revealed clinically relevant sensitizations in some patients, with subsequent improvement upon allergen avoidance. Importantly, patch test reactivity is preserved during dupilumab therapy, allowing for reliable diagnostic investigation [37]. Some authors have also suggested that DFR may represent a form of localized treatment failure, particularly in cases where facial involvement persists despite widespread clearance of eczema elsewhere [51]. Other potential contributors include alterations in the skin microbiome, seborrheic or rosacea-like dermatoses (e.g., *Demodex*-associated inflammation), and rare cases of hypersensitivity to dupilumab itself [37]. In a multicentre prospective cohort study, Kozera et al. aimed to identify potential predictive biomarkers for dupilumab-associated head and neck dermatitis (DAHND) by investigating the association between baseline Malassezia-specific IgE levels and the development of this AE [55]. The study enrolled 171 adult patients initiating dupilumab for moderate-to-severe AD and followed them longitudinally to monitor for new or worsening dermatitis involving the H&N. DAHND was defined as either a new-onset facial rash or a ≥50% increase in H&N EASI subscore compared to baseline. A total of 25 patients (14.7%) developed DAHND during the follow-up period [55]. Importantly, all patients affected by DAHND still achieved EASI-75, highlighting that regional flares may occur in the context of otherwise successful systemic disease control. Demographic features (age, sex), total serum IgE, and baseline EASI scores did not differ significantly between DAHND and non-DAHND groups [55]. However, a striking difference was observed in Malassezia-specific IgE levels, which were significantly elevated in DAHND patients (median: 31.95 kU/L) compared to non-affected individuals (median: 2.27 kU/L, *p* = 0.005), suggesting that pre-treatment Malassezia sensitization represents a strong and independent risk factor for DAHND [55]. In a case series by de Wijs et al., seven adult patients with moderate-to-severe AD developed sharply demarcated, erythematous lesions confined to the H&N region while on stable dupilumab therapy [56]. Notably, these reactions occurred despite overall excellent systemic disease control, often after several months of treatment (range: 10–39 weeks), and were not present at baseline in their reported form [56]. Histopathologically, lesional biopsies revealed a consistent pattern across all cases, with: ectatic superficial capillaries, perivascular lymphohistiocytic infiltrates, and psoriasiform epidermal hyperplasia in over half the samples [56]. Interestingly, there was no spongiosis, no eosinophilic or neutrophilic infiltrates, and normal expression of CD3 (T cells), CD68 (histiocytes), CD117 (mast cells), and CD20 (B cells) [56]. These findings suggest that DAHND may represent a psoriasiform drug-induced reaction, immunologically and histologically distinct from both acute and chronic AD [56]. This idea aligns with other reports suggesting that biologic therapies targeting one axis of inflammation may induce immune deviation, culminating in paradoxical dermatoses [57]. Among the many hypotheses proposed to explain DAHND, ACD to environmental allergens is gaining increasing recognition [37,52,54]. In this context, Napolitano et al. presented an insightful case series describing ACD to Compositae family plants as a potential and underdiagnosed cause of dupilumab-associated facial and neck dermatitis in AD patients [54]. The authors reported on three male patients (aged 24–56 years) with moderate-to-severe AD who had achieved marked improvement following dupilumab initiation and then developed erythematous, oedematous, and scaly dermatitis localized to the H&N area after a mean of 24 weeks of treatment [54]. These flares occurred despite the generalized improvement of AD, raising suspicion for an alternative or coexistent diagnosis. Patch testing using the SIDAPA baseline and plant allergen series revealed strong positive reactions to SLs mix in all three individuals (++ at day 2/3/4) and additional reactivity to Compositae mix in one case [54]. These findings confirmed the diagnosis of contact sensitization to Compositae, a known cause of photo-distributed ACD typically affecting exposed areas such as the face and neck [54]. From a pathophysiological perspective, the study reinforces the idea that dupilumab’s blockade of the Th2 axis may unmask Th1/Th17-driven conditions, such as ACD [37,52]. In conclusion, dupilumab has proven effective in HND, with supportive evidence from both clinical trials and real-life studies [35,36]. However, treatment responses in this region are often less pronounced than in other body sites [50]. Moreover, FR has been reported in a considerable proportion of patients in routine practice, occasionally leading to treatment discontinuation [51].

### 4.2. Tralokinumab

Tralokinumab is a fully human IgG4 monoclonal antibody that specifically targets and neutralizes interleukin-13 (IL-13), preventing its binding to the type II receptor complex (IL-4Rα/IL-13Rα1) [58]. Unlike broader cytokine inhibitors, tralokinumab does not interfere with IL-4 signalling via either the type I or type II receptors [59]. This selective inhibition allows for the preservation of upstream immune activation while effectively blocking downstream IL-13–mediated effector functions, including pruritus, tissue remodelling, and impairment of the skin barrier [60]. Beyond its effect on type 2 inflammation, recent evidence suggests that tralokinumab may indirectly modulate IL-22–mediated (type 22) pathways, which are associated with keratinocyte hyperproliferation, microbial dysbiosis, and barrier impairment, key features implicated in HND [39]. Persistent type 22 activation has been proposed as a possible contributor to DAHND [61,62]. In this context, tralokinumab’s more selective targeting of IL-13 may offer a therapeutic advantage in patients with HND. Clinical data from the ECZTRA 1 and 2 pivotal trials, as well as their long-term extension study (ECZTEND), support the efficacy of tralokinumab in treating HND [38]. In a pooled analysis including 1192 patients treated with tralokinumab 300 mg every two weeks (Q2W), 90% had baseline H&N involvement [38]. Median H&N EASI scores improved significantly from 3.0 at baseline to 0.4 at week 52 and further to 0.2 at week 152 in ECZTEND [38]. Notably, H&N erythema subscores also improved progressively, with 74.8% of patients achieving erythema grade 0–1 by week 152, compared to 10.5% at baseline [38]. A positive correlation between H&N EASI scores and DLQI was observed, including specific DLQI domains related to skin discomfort and self-consciousness, further supporting the clinical relevance of regional improvement [38]. In terms of safety, paradoxical facial erythema was reported in 1.0% (7/678) of patients with low baseline erythema scores. Among these, only one case was associated with worsening AD, which resolved with continued treatment [38]. A multicenter study involving 416 adults with severe AD evaluated the long-term efficacy of tralokinumab over 52 weeks. Patients were stratified into six clinical phenotypes, including a portrait/H&N subgroup (8.6%) [63]. Clinical outcomes assessed included EASI, P-NRS, DLQI, and ADCT. Tralokinumab led to significant improvements across all phenotypes (*p* < 0.001), with the portrait/H&N phenotype showing a robust clinical response [63]. By week 24, this subgroup was among those with the highest EASI-75 achievement rates, alongside classical and generalized lichenoid types. Improvements in pruritus and QoL were consistent across all subgroups [63]. The overall drug survival at 52 weeks was 83.2%, indicating high adherence and tolerability. AEs were infrequent and primarily included psoriasiform and ocular reactions [63]. In another study involving 129 patients treated over 36 weeks, tralokinumab effectiveness was evaluated by measuring EASI scores on four anatomical sites (head/neck, trunk, upper limbs, lower limbs) and four AD clinical signs (erythema, oedema/papulation, excoriation, lichenification) [64]. Results showed a consistent reduction in EASI scores across all body regions and clinical signs. The greatest improvement was noted on the lower limbs, while EASI-75 achievement rates at week 36 were comparable across sites (72.6–77.6%), including the head and neck [64]. Among clinical signs, excoriation showed the greatest improvement, with EASI-75 rates at week 36 for excoriation (69.4%), erythema (71.1%), lichenification (68.4%), and edema/papulation (60.5%) [64]. Based on these findings, tralokinumab demonstrates sustained efficacy in improving H&N involvement in patients with moderate-to-severe AD, including those who may not respond optimally to dupilumab. Its selective IL-13 inhibition and potential downstream effects on IL-22 pathways may explain its clinical benefit in this subset [39]. Despite rare cases of paradoxical erythema, long-term safety data support the continued use of tralokinumab in managing HND, even in challenging clinical scenarios [38].

### 4.3. Lebrikizumab

Lebrikizumab is a novel monoclonal antibody that selectively binds IL-13 with high affinity and a slow dissociation rate, thereby effectively inhibiting IL-13–mediated signalling. Its mechanism of action allows for targeted modulation of type 2 inflammation while sparing IL-4 pathways, potentially preserving broader immune homeostasis [65]. The efficacy of lebrikizumab in HND was specifically evaluated in a pooled analysis of three Phase III trials (ADvocate1, ADvocate2, and Adhere), with regional endpoints including H&N EASI subscores, erythema, and patient-reported facial dermatitis [40]. In the pooled population of ADvocate1 and 2 (N = 851), patients treated with lebrikizumab Q2W showed significantly greater improvement (assessed by the least squares mean (LSM) percentage improvement from baseline) in H&N EASI at week 16 compared to placebo (68.1% vs. 37.0%; *p* < 0.001), with meaningful effects noted as early as week 2 [40]. Erythema scores in the H&N region also significantly improved by Week 2 and were sustained through Week 16 [40]. Among patients with baseline facial dermatitis, 60.1% achieved clinical improvement or clearance, while 21.1% achieved complete clearance by week 16. These rates were both statistically superior to placebo (37.5% and 9.3%, respectively; *p* < 0.01) [40]. Similar findings were confirmed in Adhere (N = 211), where the addition of TCS to lebrikizumab further enhanced outcomes: 70.2% of patients experienced improvement or clearance of facial dermatitis, and 71.5% achieved >70% reduction in H&N EASI at week 16 [40]. Across trials, the incidence of AEs related to H&N and facial erythema was not increased in the lebrikizumab group versus placebo during the 16-week controlled period and these findings remained stable over long-term exposure with most patients treated for 52 weeks [40].

In a real-world study involving 162 patients treated for 36 weeks, disease activity was evaluated by assessing the EASI scores across four anatomical regions (head/neck, trunk, upper limbs, lower limbs) and four clinical signs (erythema, edema/papulation, excoriation, and lichenification) [41]. The treatment led to progressive reductions in EASI scores across all anatomical sites and clinical signs. By week 36, EASI-100 response rates were highest on the upper limbs (48.8%) and trunk (46.3%), while responses were slightly lower on the head/neck (28.9%) and lower limbs (33.3%). Among clinical signs, excoriation showed the most substantial improvement, with an EASI-100 response of 60.6%, outperforming erythema (35.3%), oedema/papulation (38.2%), and lichenification (37.1%) [41]. In conclusion, lebrikizumab demonstrates robust efficacy and a favourable safety profile in the treatment of HND, with rapid and sustained improvement in regional signs, including erythema and facial dermatitis. Importantly, no increased risk of HND or facial AEs was observed, even with prolonged exposure [40,41]. These findings support lebrikizumab as a safe and effective therapeutic option, particularly in patients with DAHND.

### 4.4. JAK Inhibitors

JAK inhibitors (JAKi) are small molecules that target the JAK-STAT signalling pathway by inhibiting the activity of Janus kinases, key enzymes involved in cytokine signal transduction from the cell surface to the nucleus [66]. By blocking these enzymes, JAKi disrupts the inflammatory cascade, leading to a reduction in hallmark symptoms of AD, including pruritus and skin lesions [67]. Currently, three JAKi are approved for the treatment of AD: abrocitinib, a selective JAK1 inhibitor; baricitinib, which inhibits both JAK1 and JAK2; and upadacitinib, a selective and reversible JAK1 inhibitor [68]. Although European guidelines do not recommend one systemic agent over another based on clinical phenotype, emerging evidence suggests that treatment response may vary among AD subtypes [69]. Accumulating clinical trial evidence and real-world data suggest that JAKi may provide superior efficacy in patients with involvement of difficult-to-treat areas, including the face, neck, hands, and genitalia, regions where biologics such as dupilumab have shown reduced effectiveness [50]. According to the expert panel of a recent consensus Delphi, Jaki is indicated as a first-line option for patients with significant disease activity in these sensitive areas [43]. Furthermore, agents such as baricitinib, upadacitinib, and abrocitinib demonstrated a rapid onset of action, marked reduction in pruritus, and a favourable safety profile, making them well suited for itch-dominant phenotypes (itch-NRS ≥ 7, BSA ≤ 40%) [43]. The consensus also acknowledged the potential utility of JAKi in complex AD presentations, such as those overlapping with psoriasis, and in patients with comorbid asthma or allergic rhinitis, though with appropriate caution [43].

A post hoc analysis conducted by Thyssen et al. showed that upadacitinib 30 mg, compared to dupilumab, resulted in a decrease in the EASI score in all body regions [70]. Specifically, the reduction in erythema of the H&N begins as early as the first week of treatment, and the Head and Neck Patient Global Impression of Severity (HN-PGIS) was reported by patients as absent or minimal from week 1 to week 24 of observation [70]. In addition, the achievement of EASI 75 in the H&N areas appears to be more durable over time than in other body areas [70]. Real-life evidence has begun to shed light on the efficacy of upadacitinib in patients with AD involving the H&N area who experience inadequate response or paradoxical worsening during dupilumab treatment [42]. In this context, Yang et al. conducted a case series involving six patients (three male, three female; mean age ± SD: 18.67 ± 7.7 years, range 12–35) with a long-standing history of AD (mean disease duration ± SD: 12.5 ± 3.5 years) who had received dupilumab for 14–20 weeks without achieving satisfactory improvement in facial and neck lesions, despite marked clearance on the trunk and limbs [42]. All patients were subsequently treated with oral upadacitinib 15 mg once daily for 12 weeks, with disease severity and patient-reported outcomes assessed at baseline and at weeks 4, 8, and 12 [42]. The most compelling data report a significant reduction in the EASI score specific to the H&N region, from a mean value of 8.1 to 0.73 at 12 weeks [42]. Similarly, pruritus severity, as measured by the P-NRS, decreased from a mean of 6.83 to 0.0, and quality of life, assessed via the DLQI, improved from 12.5 to 0.67 over the same period [42]. These findings are further supported by another real-world experience by Gori et al., in which 6 out of 31 patients treated with dupilumab, who exhibited persistent dermatitis in sensitive areas, including the H&N, were switched to upadacitinib 15 mg after a 12-week washout period [71]. A rapid and substantial clinical response was observed, with the mean baseline EASI score of 17.3 dropping to 1.2 after just 4 weeks of treatment [71]. Abrocitinib has shown encouraging results in both real-world settings and randomized controlled trials. A post hoc analysis of the phase 3 JADE COMPARE study provided important insights into the regional efficacy of abrocitinib [72]. In this trial, adult patients with moderate-to-severe AD were randomized to receive abrocitinib 200 mg or 100 mg once daily, dupilumab 300 mg every two weeks, or placebo, all in combination with background topical therapy. Notably, over 96% of participants presented with H&N involvement at baseline [72]. Abrocitinib 200 mg demonstrated a rapid and robust response in the H&N region, achieving a median time to EASI-75 of 29 days and EASI-90 of 57 days, both significantly faster than abrocitinib 100 mg (57 and 110 days, respectively) and dupilumab (57 and 112 days, respectively) [72]. At week 2, LSM percentage reduction in EASI score for the H&N region was −52.5% with abrocitinib 200 mg, compared to −51.3% with abrocitinib 100 mg and −41.3% with dupilumab; improvements were sustained through week 16, reaching −84.5%, −72.4%, and −82.3%, respectively [72]. Moreover, abrocitinib induced significant reductions across all regional EASI subscores, including erythema, induration/papulation, excoriation, and lichenification, as well as SCORAD extent and intensity items, particularly pruritus and sleep loss [72]. Real-life data also support abrocitinib effectiveness in the management of HND, as demonstrated by Napolitano et al. in a multicentre study involving 78 patients with AD in sensitive areas and treated with abrocitinib or baricitinib [73]. The mean baseline EASI score for the H&N area was 2.29 for patients treated with abrocitinib and 2.38 for patients treated with baricitinib [73]. At week 16, a mean EASI of 0.38 was reported for abrocitinib and an EASI of 0.18 for baricitinib. Furthermore, no new cases of AD in the area were reported during the entire observation period [73]. This observation is further supported by case-based real-life data reported by Santosa and Yew, who documented the clinical course of two patients with DAHND that proved refractory to conventional topical and systemic agents, including baricitinib [74]. In both cases, the onset of HND occurred within weeks of initiating dupilumab and persisted despite adjunctive antifungal therapy and discontinuation of the biologic. Notably, transition to abrocitinib 200 mg daily resulted in rapid and sustained clinical improvement, with visible resolution of facial lesions within four weeks and disease control maintained over a six-month follow-up [74]. Baricitinib has shown efficacy in itch-dominant phenotypes, especially in patients with a body surface area (BSA) involvement of less than 40% and a P-NRS score greater than 7 [75]. A phase 3 trial comparing the efficacy of baricitinib 4 mg with placebo in different areas of the body showed that 58.3% of patients in the H&N area achieved EASI-75 at week 16 of observation [75]. A recent Delphi consensus among Italian experts reaffirmed the expanding role of JAKi in the management of moderate-to-severe AD, particularly in complex clinical scenarios [43].

## 5. Conclusions

HND remains a clinically challenging phenotype of AD, often associated with a significant impact on patients’ quality of life and psychological well-being [8,9,13]. While biologic therapies such as dupilumab, tralokinumab, and lebrikizumab have demonstrated substantial efficacy in treating moderate-to-severe AD, accumulating evidence from randomized clinical trials and real-world studies suggests that their therapeutic effect in the HND region may be attenuated compared to other anatomical sites [50]. In particular, dupilumab, despite its well-established role in AD management, has been associated with paradoxical FR and persistent or new-onset HND in a subset of patients [37,51,52]. The emergence of FR during anti–IL-4/IL-13 therapy underscores the need for careful differential diagnosis (ACD, Malassezia hypersensitivity, alcohol-induced facial flushing) and tailored therapeutic interventions ranging from topical agents to systemic switches [51,52,54].

JAKi have recently emerged as a promising alternative, particularly in patients with difficult-to-treat HND, rapid symptom onset, or dupilumab-associated FR. Recent expert consensus supports their use as a first-line option in selected phenotypes, given their rapid onset of action, broad anti-inflammatory profile, and potential to address IL-22–driven inflammation [43].

In conclusion, optimal management of HND in AD requires a personalized, phenotype-driven approach that incorporates accurate diagnostic workup, therapeutic flexibility, and long-term monitoring to ensure both efficacy and safety across diverse patient profiles.

## Figures and Tables

**Table 1:** Comparative overview of systemic treatments for Head and Neck Dermatitis (HND).

Treatment	Mechanism	Evidence in HND (Key Data)	Facial/HND Adverse Events	Notes
**Dupilumab**	IL-4Rα blockade (IL-4/IL-13 inhibition)	Moderate efficacy; CHRONOS H&N erythema LS mean −65% [35]; real-life CR 30–55% [36]	Facial redness 4–43.8% [37]	Most studied; paradoxical erythema possible [35,36,37]
**Tralokinumab**	Selective IL-13 blockade	EASI-H&N improved from 3.0 → 0.4 at week (W) 152 [38]; erythema grade 0–1 in 75% at W152 [38]	Rare paradoxical facial erythema (<1%) [38]	Potential advantage in IL-22-driven phenotypes [39]
**Lebrikizumab**	High-affinity IL-13 blockade	H&N EASI −68.1% vs. −37% placebo (W16); 60.1% improvement in facial dermatitis [40]	No increased facial AEs [40]	Strong erythema and facial dermatitis improvement [40,41]
**JAK inhibitors (upadacitinib, abrocitinib, baricitinib)**	JAK1/JAK2 inhibition (varies by agent)	Superior regional efficacy; upadacitinib reduced H&N EASI 8.1 → 0.73 [42]; rapid clearance even after dupilumab FR	Acneiform eruptions possible	Best for rapid response or dupilumab-FR [43]

## Data Availability

Data that support the findings of this study are available from the corresponding author upon reasonable request.
